# Low Error Kramers-Kronig Estimations Using Symmetric Extrapolation Method

**DOI:** 10.2478/joeb-2021-0017

**Published:** 2021-12-27

**Authors:** G.A. Ruiz, C.J. Felice

**Affiliations:** 1 Laboratorio de Medios e Interfases, Departamento de Bioingeniería, FACET-UNT; INSIBIO-CONICET, Argentina

**Keywords:** Kramers-Kronig, symmetric, extrapolation

## Abstract

Kramers-Kronig (KK) equations allow us to obtain the real or imaginary part of linear, causal and time constant functions, starting from the imaginary or real part respectively. They are normally applied on different practical applications as a control method. A common problem in measurements is the lack of data in a wide-range frequency, due to some of the inherent limitations of experiments or practical limitations of the used technology. Different solutions to this problem were proved, such as several methods for extrapolation, some of which based on piecewise polynomial fit or the approach based on the expected asymptotical behavior. In this work, we propose an approach based on the symmetric extrapolation method to generate data in missing frequency ranges, to minimize the estimated error of the KK equations. The results show that with data from impedance measurements of an electrode-electrolyte interface, the adjustment error of the transformed functions can be drastically reduced to below 1%.

## Introduction

It is well known that the real and imaginary parts of certain complex function R(ω)are related to each other by the KK equations. However, this is only possible if the complex function $R(\omega )=R^{\prime }(\omega )+jR^{\prime \prime }(\omega )$represents the Fourier transformation of a linear and causal physical process. That is, if a stimulus s(t)is applied to a specific linear physical system, its temporal response r(t)is related to the stimulus applied to an ordinary differential equation with constant coefficients. So, if the stimulus is real, the response will also be real. That system can also be analyzedin the frequency domain. The link between both domains is given by the Fourier transformation:


(1) $$R(\omega )=\int_{-\infty }^{\infty }r(t)e^{-j\omega t}dt$$

where ω=2πf  and fis the frequency in Hz.

Kramers [1] and Kronig [2] developed the equations that bear their names. When working on linear optical spectroscopy, they can be applied to a large number of physical processes and are one of the most general relationships in electrodynamics. KK relationships are established between the real part of certain quantities that characterize the physical dispersion processes (dielectric permittivity, magnetic permeability, conductivity, etc.) and the imaginary part of these quantities that characterize the physical absorption processes.

Let $Z(\omega )=Z^{\prime }(\omega )+jZ^{\prime \prime }(\omega )$be the electrical impedance of a material medium between two metallic electrodes, where are real functions. If we know $Z^{\prime \prime }(\omega )$and the value of $Z^{\prime }(\omega )\text{ for }\omega \to \infty ,\text{ KK}$equations allow us to obtain $Z^{\prime }(\omega )$as shown in equation (2).


(2) $$Z^{\prime }(\omega )=Z^{\prime }(\infty )+\left(\frac{2}{\pi }\right)\int_{0}^{\infty }\frac{xZ^{\prime \prime }(x)-\omega Z^{\prime \prime }(\omega )}{x^{2}-\omega^{2}}dx$$

If instead of $Z^{\prime }(\infty ),$

we know $Z^{\prime }(0),$then equation (3) allows us to obtain $Z^{\prime }(\omega )$for any values of the angular frequency ω.


(3) $$Z^{\prime }(\omega )=Z^{\prime }(0)+\left(\frac{2\omega }{\pi }\right)\int_{0}^{\infty }\frac{\left(\frac{\omega }{x}\right)Z^{\prime \prime }(x)-Z^{\prime \prime }(\omega )}{x^{2}-\omega^{2}}dx$$

On the other hand, if we know Z′(ω), equation (4) allows us to obtain Z′′(ω).


(4) $$Z^{\prime \prime }(\omega )=-\left(\frac{2\omega }{\pi }\right)\int_{0}^{\infty }\frac{Z^{\prime }(x)-Z^{\prime }(\omega )}{x^{2}-\omega^{2}}dx$$

If Z′′(ω) has the formula:


(5) $$Z^{\prime \prime }(\omega )=\frac{a\;\omega }{1+b\;\omega^{2}}$$

The integrals (2) and (3) give as a result:


(6) $$\int_{0}^{t}\frac{xZ^{\prime \prime }(x)-\omega Z^{\prime \prime }(\omega )}{x^{2}-\omega^{2}}\;dx=\frac{a\;arctan(\sqrt{b}\;t)}{\sqrt{b}\left(1+b\;\omega^{2}\right)}$$

(7) $$\begin{array}{l} \int_{0}^{t}\frac{\left(\frac{\omega }{x}\right)Z^{\prime \prime }(x)-Z^{\prime \prime }(\omega )}{x^{2}-\omega^{2}}\;dx\\ \;=-\frac{a\sqrt{b}\;\omega \;arctan(\sqrt{b}\;t)}{\left(1+b\;\omega^{2}\right)} \end{array}$$

If now Z′(ω) is expressed in terms of ω as shown in equation (8)


(8) $$Z^{\prime }(\omega )=c+\frac{d}{1+b\;\omega^{2}}$$

The integral (4) gives as a result:


(9) $$\int_{0}^{t}\frac{Z^{\prime }(x)-Z^{\prime }(\omega )}{x^{2}-\omega^{2}}\;dx=-\frac{d\sqrt{b}\;arctan(\sqrt{b}\;t)}{\left(1+b\;\omega^{2}\right)}$$

In many situations, especially when conducting experiments, a limited number of measured values of Z′ and Z′′ are available in a narrow range of frequencies. This limited frequency range avoids knowing ω →∞ or ω → 0, limiting the validity of the KK equations. The limitation may have different origins, for example, it may be purely technological, such as the Solartron 12508W or HP 4291 impedance analyzers, which have ranges from 10 μHz to 65 kHz and 1 MHz to 1.8 GHz respectively.

A frequency range is also limited when separating a phenomenon of interest from others, reducing the range of measured frequencies, as in the monitoring of α, β and δ dispersions of biological suspensions. For example, all the information needed to characterize the β dispersion of a yeast suspension from an industrial fermenter is in the range of 0.1 MHz to 10 MHz [3]. Other dispersions outside this range can also be observed, but they are not of practical interest. The necessary information to monitor yeast growth is only in the mentioned range.

In the case of yeasts, real and imaginary components for ω → ∞ and ω → 0 can be obtained, simply by assuming that the values outside the working range remain constant. This leads us to assume that there are measurements

between 0 and ∞. This is not the case when impedance is used to monitor electrochemical sensors. Here, the ranges of frequency to observe the behavior of the electrode-electrolyte interfaces must include very low frequencies, difficult to measure experimentally.

For example, to complete the sigmoid curve of interface impedance modelled with the Randles circuit, it is necessary to measure frequencies lower than 0.1 Hz [4]. This is impractical because it is very difficult to keep stable measurements for enough time to complete a cycle at very low frequencies, where a cycle can take several minutes.

All these limitations of information that Z′ and Z′′ give us, play an important role when using KK equations to verify that the system of measurement is working correctly.

Several works about extrapolating experimental results below the frequency at which the imaginary part of the impedance presents the maximum have already been published.

These methodologies are based on the symmetry of the imaginary impedance around the frequency of the maximum and are limited to systems having one time constant. Kendig and Mansfeld [5], for example, obtained the polarization resistance from the following equation:


(10) $$\begin{array}{l} R_{p}=Z^{\prime }(0)-Z^{\prime }(\infty )\\ =\left(\frac{4\ln(10)}{\pi }\right)\left|\int_{-\infty }^{\log\left(\omega_{m}\right)}Z^{\prime \prime }(\omega )\;d\log_{10}(\omega )\right| \end{array}$$

Macdonald and Urquidi-Macdonald [6] presented a method of experimental data extrapolation consisting of polynomial fit. The authors evaluated the integral in equation (11) by segments, adjusting the experimental data of Z′′ versus frequency with a fifth-order polynomial, given by equation (12), using the least squares technique.


(11) $$\begin{array}{rl} & R_{p}\approx \left(\frac{2}{\pi }\right)\int_{x_{\min}}^{x_{\max}}\frac{Z^{\prime \prime }(x)}{x}\;dx \end{array}$$

(12) $$\begin{array}{r} Z^{\prime \prime }(x)=\sum_{i=0}^{5}a_{i}x^{i} \end{array}$$

Equation (11) follows from equations (2) and (3).

The segments on which the integral is evaluated are chosen to coincide with the sign of Z′′ changes or with changes in the gradient of Z′′, This methodology of experimental data extrapolation is also presented in ref [7].

Esteban and Orazem [8] proposed an approach that avoids the inconveniences derived from extrapolations with polynomials and also does not require making assumptions about asymptotical behaviors. Authors use an algorithm to determine the functions of the real and imaginary impedance components in a region of ω for which no experimental data are available. Then, for each frequency, the polynomial functions of K and M order given by

equations (13) and (14) are replaced in equations (4) and (2) respectively.


(13) $$\begin{array}{rl} & Z_{r}(\omega )=\sum_{k=0}^{K}a_{k}^{\left(1\right)}{\left(\log\omega \right)}^{k} \end{array}$$

(14) $$\begin{array}{rl} & Z_{i}(\omega )=\sum_{m=0}^{M}a_{k}^{\left(2\right)}{\left(\log\omega \right)}^{m} \end{array}$$

Due to the complicated form of the integrands, the integrals were numerically solved.

Another approach shown in the literature is based on the expected asymptotical behavior of a typical electrochemical system [9].

That is, to extrapolate the Z′′(ω) value to ω = 0, it is assumed that the function is proportional to ω, when ω → 0, This is consistent with the behaviour in frequency of Z′′(ω) in a Randles circuit.

For its part, the real part of the impedance tends to a constant value, which is the sum of the electrolytic solution resistance and the charge transfer resistance. The last resistance models the non-linearity of the electrode-electrolyte interface.

Finally, it should be mentioned that the lack of agreement between experimental data and the corresponding KK transformations can be attributed to two factors: on the one hand, the questions associated with the non-linearity or non-causality of the system and, on the other hand, the questions related to the low precision of the measurement systems at a very low frequency.

In order to overcome those limitations, we propose to extrapolate data outside of the available range of measurement, significantly improving the results of applying the KK transformation. The method, which is based on the symmetry of Z′′(ω), can be applied in impedance measurements of the electrode-electrolyte interface (EEI), when the expected behavior responds to the Randles model.

## Materials and methods

### Electrochemical cell

In order to illustrate the application of the KK transformation and validate the method, measurements were made using a tripolar cell in an electrolytic solution.

**Fig.1:**
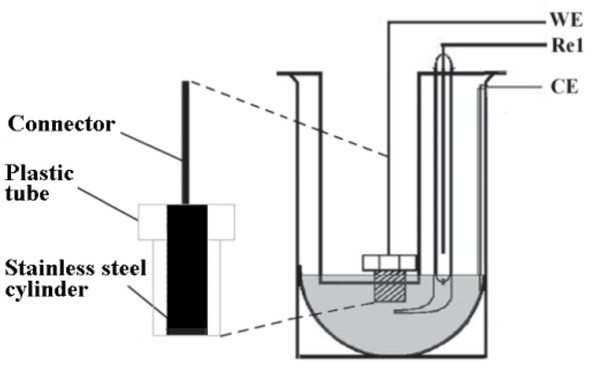
Three-electrode cell. WE: working electrode, Re1. reference electrode, CE: counter-electrode.

Several frequency sweeping experiments were carried out at constant overpotential and using electrodes polished with sandpaper #180, The three-electrode electrochemical cell used (Fig. 1) is composed of a working electrode (WE) and an Ag/AgCl reference electrode (Re1). An AISI 304 stainless steel concave counter-electrode (CE) 85 mm was also used. The WE is a solid cylinder (1.5 cm long) made also of AISI 304 stainless steel with only 1 cm^2^ of its transversal section exposed; the rest was insulated with Grilon. The CE area was larger than the working electrode to minimize its impedance. The sampling frequency was such that the measurement points were equally spaced in a logarithmic scale taking 5 points per decade. The integration time of the measurements was 1 cycle for frequencies lower than 6.28 rad/s and 16 cycles for the rest.

In every case, the potential was stabilized in an open circuit until the voltage shift was lower than 0.05 mV/seg. Then, the equivalent series resistance and reactance measurements were carried out. The overpotentials used were 10 mV. The electrolyte solution used was NaCl 0.9%.

Electrochemical measurements were performed with a Solartron 12508W Impedance Analyzer composed of a Solartron 1287 Electrochemical Analyzer and a Solartron 1250 Frequency Response Analyzer, commanded by the software provided by the manufacturer (ZPlot®, Scribner Associates Incorporated).

### The symmetric data extrapolation method

The method consists of expanding the amount of data available in a range of frequencies greater than that measured, extrapolating data from the available measurements.

The method requires two conditions for its application: (i) the curves of Z′(ω) and Z′′(ω) must be symmetric respect to the central frequency, a condition that is fulfilled by dispersions with a single time constant, and (ii) the frequency range of the measurements must cover at least the central frequency of the dispersion until the percentage change of Z′(ω) is below 0.1, Extrapolation allows us to reduce the difference between the curves of the measured and the calculated data using the KK transformation.

Extrapolation is performed at low frequency, from the lowest limit of frequency ωmin up to the central frequency of the dispersion ωp, taking into account that Z′(ω) − Z′(∞) is symmetric respect to ωp , This frequency ωp is also the frequency in which Z′′(ω) is maximum. ωp , often called the peak frequency.

In this work, we used $\omega_{p}$to locate the point of $Z^{\prime }(\omega ),$It is where its concavity changes, as schematically shown in Figure 4.

**Fig.2:**
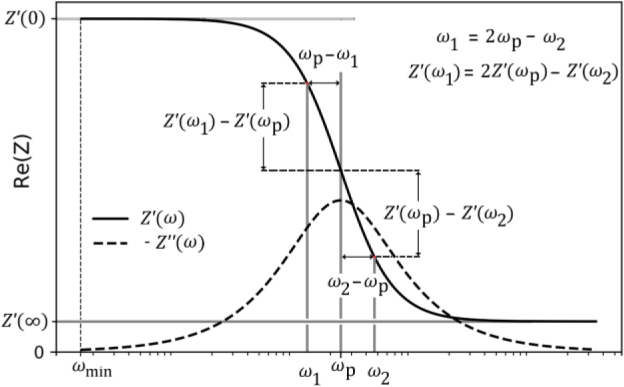
Frequency and amplitude of the interpolated points. $\omega_{p}:$central frequency of the dispersion.

**Fig.3:**
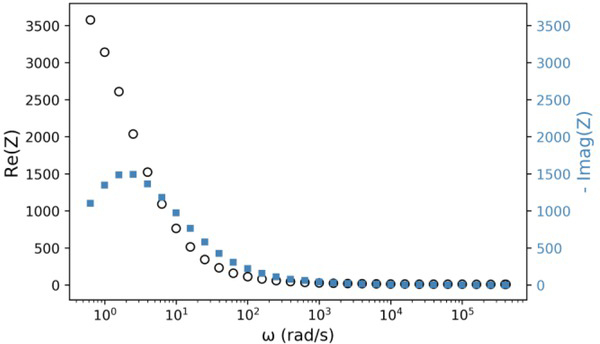
Resistance (Black) and reactance series equivalents (Steel Blue) of the EEI versus frequency for AISI 304 stainless steel electrode polished with sandpaper #180 in NaCl 0.9% solution. Overpotential applied = 10 mV.

**Fig.4:**
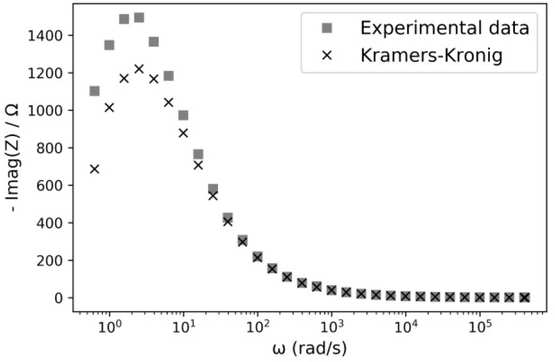
Reactance series equivalent of the ZEEI versus frequency. Experimental data (◼) and applying the KK equation (4) to the experimental data (**x**).

Figure 2 graphically shows how to extrapolate frequency and amplitude of $Z^{\prime }(\omega )$for points before $\omega_{p}.$The corresponding equations are:


(15) $$\begin{array}{r} \omega_{1}=2\omega_{p}-\omega_{2} \end{array}$$

(16) $$\begin{array}{r} Z^{\prime }\left(\omega_{1}\right)=2Z^{\prime }\left(\omega_{p}\right)-Z^{\prime }\left(\omega_{2}\right) \end{array}$$

### Ethical approval

The conducted research is not related to either human or animal use.

## Results

Figure 3 shows the plots of $Z^{\prime }(\omega )\text{ and }Z^{\prime \prime }(\omega )$versus frequency for AISI 304 stainless steel in NaCl 0.9% solution between 0.628 and 4.084 ∙ 10^5^ (rad/s).

Equation (4) solved numerically is used to obtain $Z^{\prime \prime }(\omega )$from $Z^{\prime }(\omega )$as shown in Figure 4, A difference of more than 20% between the measured and calculated data at $\omega_{p}=$$2.04\;(rad/s)\;is\;observed.\;Z_{p}^{\prime \prime }=Z^{\prime \prime }\left(\omega_{p}\right)$is the maximum of $Z^{\prime \prime }\text{ at }\omega =\omega_{p}.$

**Fig.5:**
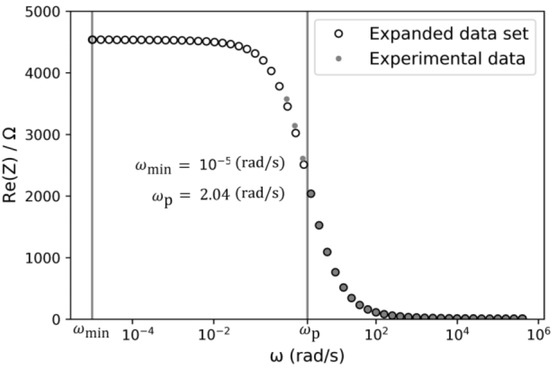
Resistance series equivalent of the EEI impedance. Expanded dataset (ο) and Experimental data (∙).

Figure 5 shows the expanded dataset of $Z^{\prime }(\omega )$after applying the proposed extrapolation method. In it, the interpolated frequency range is indicated with vertical lines $(\omega_{min}\;to\;\omega_{p})$.

**Fig.6:**
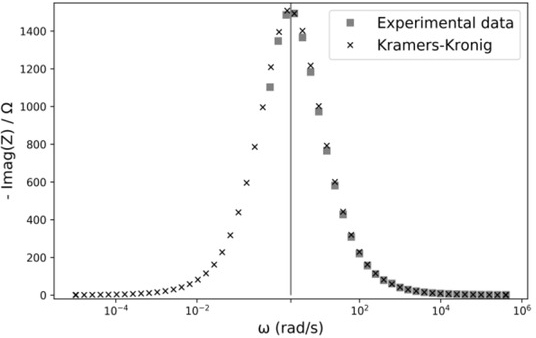
Reactance series equivalents of the EEI versus frequency. Experimental data (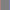) and applying the KK equation (4) to the expanded dataset (**x**).

Figure 6 shows the result of applying the KK equation (4) again, but this time to the expanded dataset.

**Fig.7:**
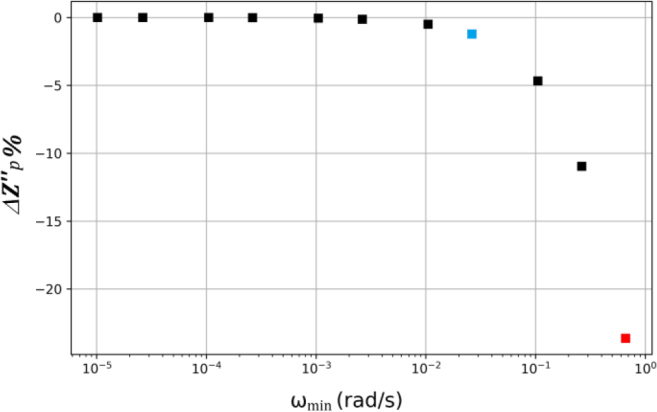
Percentage change of $Z_{p}^{\prime \prime }\left(\Delta Z_{p}^{\prime \prime }\%\right)\text{ versus }\omega_{min}.$Red point: case without extrapolation (figure 4). Blue point: $\Delta Z_{p}^{\prime \prime }\%=-1.$


It should be noted that the agreement between maximum of $Z^{\prime \prime }(\omega )$experimental data and the result obtained using the KK equation (4) depends on the lower frequency limit $\omega_{min}.$To analyse this aspect in more detail, Figure 7 shows the percentage change of $Z_{p}^{\prime \prime }\;\left(\Delta Z_{p}^{\prime \prime }\%\right)$versus ω_min_ , $\Delta Z_{p}^{\prime \prime }\%$is calculated with equation (17).

(17) $$\begin{array}{l} \Delta Z_{p}^{\prime \prime }\%=\left[Z_{p}^{\prime \prime }\left(\omega_{\min}\right)-Z_{p}^{\prime \prime }(\exp)\right]\\ \;\cdot 100/Z_{p}^{\prime \prime }(\exp) \end{array}$$

## Discussion and conclusions

Ideally, equations (2) through (4) of KK allow us to obtain $Z^{\prime }(\omega )\text{ from }Z^{\prime \prime }(\omega )$and vice versa. In practice, the range of measurement frequencies must be enough to obtain an acceptable error within the required application. Normally, electrochemical measurement systems cover the range of high frequencies $\left(>\omega_{p}\right)$quite well and the measurements are quite precise. For example, Solartron 1250 in combination with the 1287 electrochemical interface allows performing electrochemical impedance measurements with an error less than 0.3% between 10 μHz and 10 kHz. However, in the low frequencies range $\left(<\omega_{p}\right),$it is very difficult, since it requires a long measurement time and it is difficult to keep the stability of experimental conditions. From the analysis presented, it is evident that the quality of the results obtained by application of the KK relations, $Z^{\prime \prime }(\omega )\text{ from }Z^{\prime }(\omega )$in the example presented, depends on the quality of the available experimental dataset and measurement errors. The extrapolation method of missing data presented in this work, as in several published works [3, 5, 8, 10], assume symmetry of the real and imaginary components of the impedance Z(ω)in the frequency domain and a system with a single time constant.

These restrictions are fulfilled in many physical and biophysical systems for certain frequency ranges.

However, unlike published works, the method presented does not use polynomial fit or assumptions about asymptotic behaviors. For frequencies lower than ω_p_, the method replicates the behaviour observed for $\omega >\omega_{p}.$This simple fact, described in Figure 2, allows us to extrapolate enough points to comply with the requirements needed to apply the KK equations.

A plot of measured data and its corresponding KK transformations using equation (4) are shown in Figure 4, It can be observed a mismatch due to insufficient measurement range.

Figure 7 shows how the theoretical adjustment depends on the amplitude of the extrapolated frequency range, by representing the percentage change of $Z_{p}^{\prime \prime }$as a function of the lower limit of the extrapolated dataset $\omega_{\min}$It can be seen that the worst case (red point) corresponds to an error of 24% when using only measured data. On the other hand, when extrapolating data at frequencies lower than $\omega_{min}=$$10^{-2}rad/s$(blue point) the error dropped below 1%.

In Figures 5 and 6 data were extrapolated up to $\omega_{min}=$$10^{-5}rad/s$, achieving an error less than 0.2%.

In conclusion, we present a simple method to extrapolate impedance data sets from linear, causal systems and with a single time constant. The new method allows us to apply the KK equations with a minimum estimation error of the real or imaginary parts of impedance.
